# Community-Acquired Antimicrobial Resistant Enterobacteriaceae in Central America: A One Health Systematic Review

**DOI:** 10.3390/ijerph17207622

**Published:** 2020-10-19

**Authors:** Lauren O’Neal, Danilo Alvarez, Renata Mendizábal-Cabrera, Brooke M. Ramay, Jay Graham

**Affiliations:** 1 School of Public Health, University of California, Berkeley, CA 94720, USA; lauren_oneal@berkeley.edu; 2 Center for Health Studies, Universidad del Valle de Guatemala, Guatemala City 01015, Guatemala; dalvarez@ces.uvg.edu.gt (D.A.); rmendizabal@ces.uvg.edu.gt (R.M.-C.); bramay@uvg.edu.gt (B.M.R.); 3 Paul G. Allen School for Global Animal Health, Washington State University, Pullman, WA 99164, USA

**Keywords:** antimicrobial resistance, Enterobacteriaceae, Central America, One Health

## Abstract

Community-acquired antimicrobial resistant Enterobacteriaceae (CA-ARE) are an increasingly important issue around the world. Characterizing the distribution of regionally specific patterns of resistance is important to contextualize and develop locally relevant interventions. This systematic review adopts a One Health framework considering the health of humans, animals, and the environment to describe CA-ARE in Central America. Twenty studies were identified that focused on antimicrobial resistance (AMR) in Enterobacteriaceae. Studies on CA-ARE in Central America characterized resistance from diverse sources, including humans (*n* = 12), animals (*n* = 4), the environment (*n* = 2), and combinations of these categories (*n* = 2). A limited number of studies assessed prevalence of clinically important AMR, including carbapenem resistance (*n* = 3), third generation cephalosporin resistance (*n* = 7), colistin resistance (*n* = 2), extended spectrum beta-lactamase (ESBL) production (*n* = 4), or multidrug resistance (*n* = 4). This review highlights significant gaps in our current understanding of CA-ARE in Central America, most notably a general dearth of research, which requires increased investment and research on CA-ARE as well as AMR more broadly.

## 1. Introduction

Antimicrobial resistant Gram-negative bacteria belonging to the Enterobacteriaceae family pose a significant risk to human health, and are categorized through identification of priority groups as “critical”, “high” and “medium” by the World Health Organization (WHO) [1]. The spread of community-acquired antimicrobial resistant Enterobacteriaceae (CA-ARE) is an increasing problem worldwide and is particularly problematic in low- and middle-income countries (LMICs) with fewer surveillance and regulation mechanisms [2]. As CA-ARE are detected in a broader geographic landscape with increasing prevalence, it has become clear that coordinated research and antimicrobial resistance (AMR) mitigation efforts should include spread in communities outside of clinical settings [3]. Understanding the mechanisms and risk factors driving CA-ARE proliferation is fundamental to prevention and control efforts.

CA-ARE are attributed to many drivers, including antimicrobial misuse in humans and animals that create selective pressure for drug resistance, as well as poor water and sanitation systems that allow for the organisms to spread. In human health, the improper use of antimicrobials leads to a selection of CA-ARE [4]. Outside of clinical settings, there is evidence that antimicrobials are misused in food animal production, where they are administered to both commercial [5] and small-scale food animals to promote growth, prevent disease and improve feed conversion efficiency [6]. Persistence and spread of CA-ARE contribute to the global problem and numerous studies have identified that travel to LMICs is a risk factor for CA-ARE [7]. CA-ARE are also likely to be spread from human and agricultural waste which are often disposed of in the environment and subsequently contaminate waterways, soil, edible crops and wildlife. Furthermore, waste from pharmaceutical manufacturing, hospitals, and a variety of industries contribute to the spread of CA-ARE [8]. The combined capability for CA-ARE to be spread outside of healthcare facilities, as well as the intra- and inter-species transfer of resistance, and the already serious state of antimicrobial resistance in Enterobacteriaceae make this family of bacteria a critical target for research [9].

Given the interconnecting factors contributing to increasing CA-ARE, understanding and responding to this issue necessitates the use of a One Health framework, which focuses on issues that occur at human, animal, and environmental interfaces [10,11]. Upholding this principle, the WHO Plan to address global AMR uses a One Health approach and asks members to follow by example when creating country-specific action plans [12]. 

This review of CA-ARE focuses on Central America, a region composed of seven countries: Belize, Costa Rica, El Salvador, Guatemala, Honduras, Nicaragua, and Panama, which are in varying stages of development in terms of demographics, health and economics (Table S1). There is evidence that the most widely available antibiotics in the Central American region are amoxicillin and tetracycline often sold without a prescription in corner stores [13]. Self-medication practice in the region also reflects the prevalent community use of amoxicillin and tetracycline to treat the common cold, or flu like symptoms [14]. In clinical medicine, however, phenotypic resistance profiles do not reflect these community antibiotic use practices where high rates of resistance to broad spectrum antibiotics have been shown in Guatemala [15]. In this context, there are likely to be other drivers of antimicrobial resistance related to hygiene and sanitation that play an important role in propagating bacterial resistance to broad spectrum antibiotics in the region [16].

Regional surveillance networks, global reporting systems, and scientific research provide current knowledge of AMR in Central America. All seven Central American countries are members of the Latin American Network for Antimicrobial Resistance Surveillance (ReLAVRA), which was established by the Pan American Health Organization (PAHO) and WHO in 1996, “to inform AMR prevention and control policies and interventions in the region, through the ongoing collection of reliable, comparable, and reproducible AMR data” [17]. Member countries are required to report AMR to ReLAVRA annually, though the last report published with individual country information was in 2014 [18]. In 2015, information from ReLAVRA was published by WHO in a report that discussed the WHO Region of the Americas together (North, Central, and South America) [3]. The same year, WHO initiated the Global Antimicrobial Resistance Surveillance System (GLASS) to support global efforts on antimicrobial resistance. Of all world regions, the region of the Americas had the lowest proportion of countries enrolled in GLASS, none of which were in Central America [19,20]. 

Though coordinated international reporting systems are still developing, some Central American countries have national surveillance working groups or action plans. Costa Rica, for example, developed a national 2018–2025 action plan on AMR that includes five strategic objectives aimed at increasing AMR education, enhancing surveillance, improving sanitation and antimicrobial use procedures, and investing in vaccine and drug development [21]. Guatemala’s National Network for Surveillance and Control of Antimicrobial Resistance (Red Nacional de Vigilancia y Control de la Resistencia Antimicrobiana, RedRAM) is a national AMR network of people across non-profit, governmental, and academic institutions that aims to begin a collaborative effort to improve communication across sectors. Like other Central American countries, Guatemala is working on creating action plans whose protocols are being developed by the Ministry of Health, in collaboration with the National Infectious Diseases Committee, PAHO-Guatemala and the Centers for Disease Control and Protection Central America Regional office (CDC-CAR), among others.

This systematic review identifies relevant research from the Central American region that characterizes CA-ARE from non-clinical human and animal (domestic and wild) samples, where there is high human–animal or human–environment overlap with the potential for human exposures.

## 2. Materials and Methods

We conducted a systematic literature review following the Preferred Reporting Items for Systematic Reviews and Meta-Analyses (PRISMA) guidelines [22]. PubMed was used to search for all English and Spanish articles published prior to 2020 using a set search query (see Supplementary Material). The database was queried on 10 February 2020. Results were imported into Covidence, a systematic review management software (www.covidence.org [23]), for inclusion and exclusion. Studies were included based on geographic and content criteria; research must have been conducted in Central American countries (i.e., Belize, Costa Rica, El Salvador, Guatemala, Honduras, Nicaragua, or Panama) and must have been original research focused on community-acquired AMR. After the initial exploration of the search results, inclusion criteria were narrowed to include only bacteria belonging to the Enterobacteriaceae family (e.g., *Salmonella*, *E. coli*, *Klebsiella*) because of their important role in human infections and their ability to spread AMR genes that can be acquired in the community [24,25,26].

**Figure 1:**
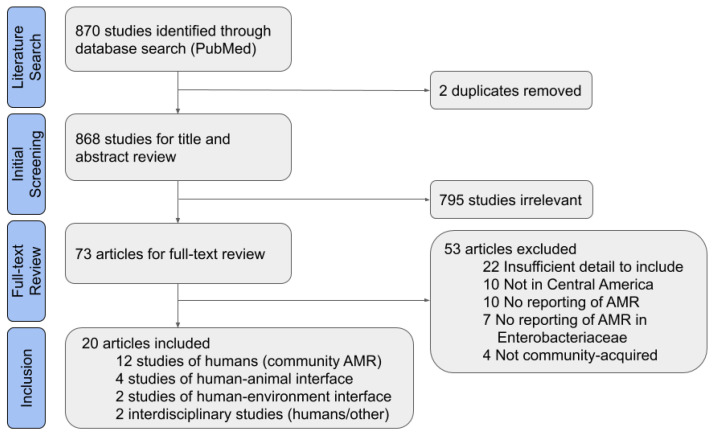
Preferred Reporting Items for Systematic Reviews and Meta-Analyses (PRISMA) Flow Diagram for systematic literature review.

Of 868 non-duplicate results, 795 studies were found to be irrelevant based on title and abstract (Figure 1). The most common reasons for exclusion were that Enterobacteriaceae were not studied, human infections were nosocomial or from an unclear source, or the study did not take place in Central America. Another 53 articles were excluded after completing the full article review. Of these, 22 studies were excluded for insufficient detail, which most commonly was due to the lack of data on the source of bacterial isolates or the species studied. Eight of the studies were excluded for insufficient detail on the isolate origin or origin of archived isolates from a biobank. Ten studies were not in Central America or focused on travelers returning from a Central American country; studies of travelers were excluded because it was unclear if the traveler also visited other countries outside of Central America. The other excluded articles did not meet the other inclusion criteria set for this review of CA-ARE, such as studying AMR or species in the Enterobacteriaceae family. 

After full-text inclusion and exclusion, 20 full texts were included for data extraction. All were reviewed for a standard set of factors, including study timeframe, study location, sample size, and type of resistance studied (Table S2). Though some of the included studies had a larger scope, only the aspects of studies relevant to CA-ARE in Central America are discussed in the results of this analysis.

## 3. Results

**Figure 2:**
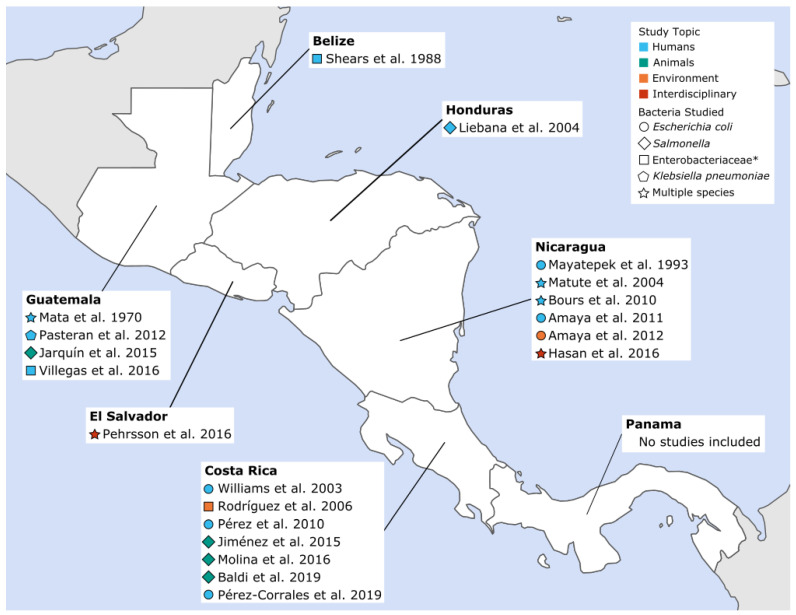
Community-acquired antimicrobial resistant Enterobacteriaceae (CA-ARE) studies included in the review by year, sample source and pathogen. * Bacterial species studied were not specifically defined beyond the family Enterobacteriaceae.

Of the 20 papers included, 12 studied community-acquired AMR in humans, four presented isolates from wildlife or animal food products, two focused on isolates from environmental samples, and two studied isolates from several categories (i.e., human and environmental samples). As shown in Figure 2, seven studies were published from Costa Rica, six from Nicaragua, four from Guatemala, and one each from Belize, El Salvador, and Honduras. No articles from Panama met the inclusion criteria for this review. More detailed information about each study, including location, sample source and size, and types of resistance studied can be found in Supplementary Materials, Table S3.

### 3.1. CA-ARE in Humans

Of the 12 studies of human isolates, 10 were categorized by sample type and symptoms experienced by study participants (e.g., diarrhea). Six studies used stool samples to investigate diarrhea [27,28,29,30,31,32], three studies collected urine samples to investigate urinary tract infections (UTIs) [33,34,35], and one investigated blood-stream infections [36]. Additionally, two were case reports of CA-ARE infection of people living in Central America [37,38]. 

While the case studies do not provide information about the prevalence of CA-ARE, they may capture sentinel AMR events. In Honduras, one report on a multidrug-resistant pediatric *Salmonella* infection was the first to demonstrate CTX-M and AmpC enzymes, which convey resistance to antimicrobials, in the Americas [37]. It was also the first report of blaCMY-2, an AMR gene, in *Salmonella* serotype Infantis, and the first report of blaCTX-M-15 in the genus *Salmonella*. Isolates from urine and stool samples were resistant to all 23 antimicrobial agents studied (see Table S3, Liebana et al. 2004 [37]) with the exception of imipenem, nalidixic acid, neomycin, tetracycline, furazolidone, and streptomycin. 

In Guatemala, *K. pneumoniae* clinical isolates from an adult patient trauma wound were the first in Latin America characterized as New Delhi Metallo-beta-lactamase-1 (NDM-1)-producing Enterobacteriaceae [38]. The *K. pneumoniae* strain was resistant to all of the beta-lactams tested, trimethoprim/sulfamethoxazole and minocycline and had reduced susceptibility to ciprofloxacin, gentamicin and chloramphenicol; it was susceptible to amikacin, nalidixic acid, levofloxacin, tigecycline, colistin and fosfomycin.

### 3.2. CA-ARE at the Human–Animal Interface

Of the four studies that took place at the human–animal interface, two focused on wildlife. In two regions of Costa Rica, intestinal samples from Asian house geckos (*Hemidactylus frenatus*, *n* = 115) were collected from geckos found in homes [39]. No *Salmonella* isolates were found to be resistant to trimethoprim–sulfamethoxazole, ciprofloxacin, or cefotaxime, which are frequently used to treat human salmonellosis in the region. Intermediate resistance to streptomycin and to sulfonamides was detected in 50% of the isolates. In raccoons (*Procyon lotor*) from urban Costa Rica, *Salmonella* was isolated from 42 (49%) of 86 viable fecal samples and were tested for antibiotic susceptibility [40]. Isolates were resistant to two antimicrobials commonly used to treat salmonellosis in Costa Rica: ciprofloxacin (9.5%) and nalidixic acid (7.1%).

The remaining two studies describe AMR in animal feed for domestic animals or in food-animal products. In Costa Rica, a study found AMR in animal feed, including poultry feed, meat and bone meal, and pet food [41]. Fewer than 8% of the *Salmonella* strains were resistant to tetracycline, and resistance profiles for serovars Anatum and Havana were highest. In Guatemala, researchers sampled 300 chicken carcasses available for purchase at a variety of retail markets and suggested that the source of the meat and storage practices have implications for AMR in raw poultry [42]. Of all the isolates analyzed, 52.4% were resistant to enrofloxacin, 40.7% to tetracycline, 37.9% to trimethoprim–sulfamethoxazole, and 35.9% to streptomycin. Resistance to trimethoprim–sulfamethoxazole was highest for isolates from supermarkets (81.8%), greater in refrigerated chickens than those stored at room temperature (66.7% versus 30.5%, respectively), and highest for samples produced by integrated poultry companies (63.0%).

### 3.3. CA-ARE at the Human–Environment Interface

Of the two studies involving environmental samples, one focused on agricultural settings. This study found that lettuce heads randomly selected from Costa Rican farms using antimicrobials on crops were contaminated with drug-resistant bacteria [43]. The proportion of Enterobacteriaceae with resistance to oxytetracycline or gentamicin ranged from 0.01% to 100% depending on the farm. A higher abundance of bacterial contamination was found on lettuce compared to samples from irrigation water from the farms.

Another study focused on AMR in *E. coli* from drinking and environmental water samples. In Nicaragua, all hospital sewage samples and 9% of well-water samples had high levels of resistance. Isolates that were resistant to antimicrobials tended to be multidrug resistant [44]. Of drug-resistant isolates from well-water, 19% were resistant to nine antibiotics: ampicillin, ceftazidime, ceftriaxone, cefotaxime, chloramphenicol, ciprofloxacin, gentamicin, nalidixic acid and trimethoprim–sulfamethoxazole. Of the multidrug-resistant hospital sewage isolates, the most common profile was ampicillin, chloramphenicol, ciprofloxacin, nalidixic acid, and trimethoprim–sulfamethoxazole. Extended spectrum beta-lactamase (ESBL) producing *E. coli* were detected in one of the three hospital sewage samples and in 26% of resistant isolates from well-water.

### 3.4. CA-ARE in Interdisciplinary Studies

Two articles studied human isolates and compared them to isolates from either animals or the environment. Though they are both examples of articles on the human–animal or human–environment interface, these studies are highlighted here because they studied humans, animals, and their environment together. Work described previously focused only on animals at a human–animal interface or environments at a human–environment interface, but not in conjunction with prevalence of CA-ARE in humans.

A study conducted in Nicaragua investigated the fecal carriage of ESBL-producing *K. pneumoniae* and *E. coli* in poultry, wild birds (Black Vultures, Cattle Egrets, White Egrets, Elegant Terns, Pigeons), and humans [45]. From each group, 100 individuals were sampled and found to have the following prevalence of ESBL-producing *K. pneumoniae* and *E. coli*: humans 27%, poultry 13%, and wild birds 8%. Isolates from wild birds and poultry were also found to have AMR genes (bla_CTX-M_) of human origin. In El Salvador, researchers conducted a metagenomics analysis, which is the untargeted sequencing of genetic material from uncultured microorganisms [46]. They characterized the resistome (i.e., all of the AMR genes in a sample) of samples from humans as well as samples from their household environment, including water, soil, animal feces, and latrines. They found that, on average, human feces and chicken coop soil resistomes shared a set of 10 antimicrobial resistance genes, more so than any other types of soil tested. In that same study, the microbial content of samples from open street sewage was more similar to samples from waste water treatment facilities than to samples from the humans living nearby, suggesting that aerobic/non-aerobic environmental changes impact bacterial content more significantly than the changing contexts in downstream sewage systems.

### 3.5. CA-ARE Across Studies

The articles included in this review studied isolates of several Enterobacteriaceae from different sources with resistance to different antimicrobials. For a more quantitative comparison, we reviewed each study for the highest percent resistance reported across classes of antimicrobials in the most prevalent bacterial species (Table 1). In studies characterizing resistance in multiple species or sample sources, the source or species with the greatest number of isolates studied were described. The antimicrobials studied as well as percent resistance reported varied significantly between studies. It is notable that later studies tend to target a single class of antibiotics, in contrast to earlier studies that examined resistance to many classes.

**Figure 3:**
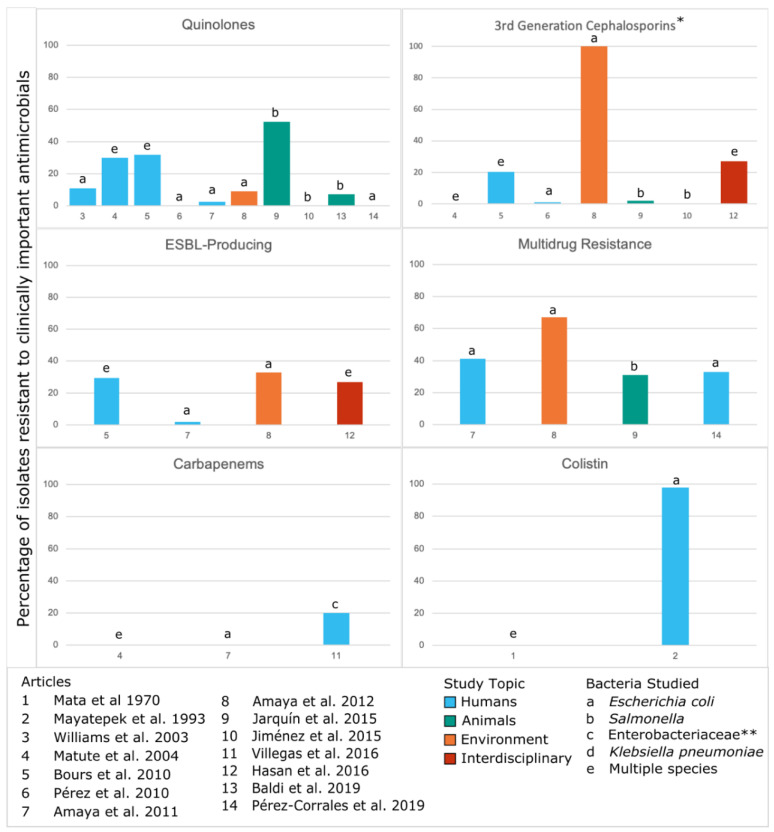
Phenotypic resistance to clinically important antimicrobials from the included studies on CA-ARE in Central America.Numbers in the x-axis correspond to the articles listed. Resistance rates are only shown for the most prevalent species as indicated in the Measure column of Table 1 though some studies tested multiple species for resistance. This figure excludes three of the articles that did not report percentages or prevalence: Liebana et al. 2004 [37] and Pasteran et al. 2012 [38] (case reports), and Pehrsson et al. 2016 [46] (resistomes). Three articles that did not study resistance to clinically important antimicrobials were also excluded from this figure: Shears et al. 1988 [32], Rodríguez et al. 2006 [43], Molina et al. 2016 [41]. In the above figure, * represents potential ESBL producers but not all studies tested for ESBL production; ** indicates that the bacterial species studied were not specifically defined beyond the family Enterobacteriaceae.

Based on the WHO Priority Pathogens [1] and the European Medicines Agency’s 2018 Categorization of Antibiotics [47], we also identified instances across studies of resistance to antimicrobials for which AMR is a major concern (Figure 3). Three studies identified high levels of resistance (>50%), including one of colistin resistance, one with third generation cephalosporin resistance and multidrug resistance, and one of quinolone resistance. Though a majority of included studies have a low-to-moderate prevalence of resistance, those with higher levels of resistance demonstrate that CA-ARE could pose a significant risk of exposure to humans.

## 4. Discussion

This review identified 20 studies of CA-ARE in Central America. There were differences in the number of studies conducted in each country; the majority of CA-ARE research was conducted in Costa Rica (*n* = 7), Nicaragua (*n* = 6), and Guatemala (*n* = 4), while Belize, El Salvador, and Honduras only had one study and Panama had none. The majority of research was in humans only (*n* = 12), while fewer studies focused on animals at the human–animal interface (*n* = 4) or the environment at the human–environment interface (*n* = 2). Two additional studies were interdisciplinary in design and included multiple One Health components (e.g., animals and human or environments and humans). Researchers have noted that there are a number of challenges in conducting One Health research from the design stage, to execution, and through to monitoring and evaluation due to the poor availability of resources and personnel, which may be why we found such a low number of One Health studies in Central America [48].

Along with differences in the number of studies conducted in each country, this review identified variations in classes of antimicrobials studied. Over time and across countries, there are many differences in which classes of antimicrobials were studied. Some identified resistance across several classes while other studies focused on only one class. When considering clinically important AMR, there were a limited number of studies on prevalence of resistance to carbapenems (*n* = 3), third generation cephalosporins (*n* = 7), colistin (*n* = 2), and multi-drug resistance (*n* = 4). Only four studies identified whether CA-ARE were ESBL-producing. Quinolones were the most frequently studied antimicrobials of clinical importance (*n* = 10). 

There were also distinct trends in rates of resistance reported in countries. High rates of resistance to aminoglycosides and sulfonamides were reported in all Guatemala, Nicaragua, and Costa Rica. By contrast, Nicaragua is the only country that reported a high percentage of resistance to cephalosporins, chloramphenicols, and polymyxins, and only Guatemala reported a high rate of resistance to quinolones. Because the studies of CA-ARE in El Salvador and Honduras were case reports and there were no CA-ARE studies identified in Panama, this review highlights that information about CA-ARE prevalence in these countries is not currently available.

In addition to highlighting a general lack of research on CA-ARE in Central America, this review found that clinical studies have limited use in a One Health framework because, without detailed information about isolate sources, it can be unclear whether clinical isolates are community-acquired AMR or hospital-acquired AMR. For example, this review excluded many studies using archived clinical isolates because they reported some source information, like sample source (e.g., urine, stool, blood), but not whether patients were outpatient or hospitalized for an extended period. Without the ability ascertain the specific source of AMR, it is difficult to determine whether the resistance measured in a study is a reflection of AMR in healthcare settings or in the community, which convey different implications for AMR in a larger One Health context. 

While the studies included in this review elucidate aspects of CA-ARE in Central America, this review was limited due to a lack of available research on the subject. One Health research was sparse. When One Health research was conducted, the bacterial species studied often did not align with bacterial species of concern in clinical settings (i.e., irrelevant to human health). All of the studies on animals in this review studied *Salmonella*, while only two studies focused on humans studied *Salmonella*. Research on CA-ARE and AMR in general needs greater uniformity in reporting and priority setting before significant comparisons can be made between AMR in clinical settings and AMR in the community, animals, and the environment.

## 5. Future Directions

Investment in local capacity will be essential for developing a One Health AMR framework in Central America. This review has shown that there are enormous knowledge gaps in the region that hinder global and regional efforts to reduce AMR spread, and international efforts, like GLASS and ReLAVRA, do not fully characterize the current AMR situation in Central America. The only country in the region with a national AMR action plan, Costa Rica, was also the country best described by existing literature on CA-ARE. It is clear that international and regional investment in understanding AMR is critical, while also improving systems that help mitigate the impact of AMR, such as healthcare access and services, patient and clinician education, expanded laboratory capacity, and regulation of antimicrobial use in all settings (clinical and agricultural). 

Major improvements to One Health surveillance of AMR are greatly needed in Central America, and there is a significant need to apply this approach with multiple sectors involved at the early stages of development. Dedicated resources for the One Health approach will likely strengthen regional capacity to prevent and control the spread of AMR by building new knowledge and understanding of the drivers for AMR locally [49]. However, to have access to more funds, to meet clear research and surveillance objectives that respond to the needs of each country and the region, it is necessary to prioritize the problem, structure strategic plans, and define specific lines of research. 

Stakeholders working in the animal, environmental and human health sectors have often found it difficult to understand each other’s objectives, and this has led to a lack of mutual understanding of the shared benefits of collaboration. Development and implementation of One Health frameworks that incorporate multidisciplinary goals and outcomes could augment this effort. Focusing on AMR that is clinically relevant (i.e., causing the greatest morbidity and mortality in hospitalized patients regionally and globally), will likely be an important start for One Health AMR surveillance programs at the early stage of development. In this review, we highlight the limited scope of information about community-acquired AMR in Central America, particularly for last-line antimicrobials like carbapenems and colistin. This review draws attention to the gaps related to the lack of studies and surveillance with the One Health approach, especially in Enterobacteriaceae [50,51,52].

The 2016 WHO global containment plan includes, among its five pillars, the improvement of knowledge of AMR through communication and education, as well as the strengthening of the scientific base through surveillance and research. Outlining the state of knowledge about AMR, especially community-acquired AMR, in Central American countries is a starting point for regional plans. This review contributes to the knowledge of this state and to the consideration of the One Health approach as a way of addressing the problem. Effective design and implementation of strategies to decrease AMR will require the consideration of risk factors encompassing interactions between humans, animals and the environment. Further, greater transparency and uniformity in data collection strategies and dissemination of results will improve cohesion and comparability of findings across the region. These efforts will require greater coordination of efforts across sectors, and among countries. 

## 6. Conclusions

This study highlighted findings on CA-ARE in Central America based on 20 studies that were conducted over the past fifty years (since 1970). The limited amount of research highlights that there is a need to strengthen multi-sectorial AMR research and surveillance. Future steps in Central America should be to strengthen the relationships among the human health, animal health and environmental sectors and create an inclusive approach to AMR, supported by professional network activities that can facilitate partnerships and create cross-disciplinary awareness and participation.

## Figures and Tables

**Table 1:** Percent of isolates resistant by antibiotic class in the included studies on CA-ARE in Central America.

References	Measure (No. of Isolates)	Aminoglycosides	Penicillin ꞵ-lactams	Cephalosporins	Carbapenems	Chloramphenicols	Polymyxins	Nitrofurans	Sulfonamides	Tetracyclines	Quinolones
Belize											
Shears et al. 1988 [32]	% resistance in Enterobacteriaceae isolates from diarrhea (ND)	0	0	ND	ND	0	ND	ND	11	22	ND
Guatemala											
Mata et al. 1970 [28]	% resistance in *Shigella dysenteriae* strains from diarrhea (*n* = 53)	98	23	0	ND	42	0	0	100	98	ND
Jarquín et al. 2015 [42]	% resistance in *Salmonella* spp. isolates from chicken carcasses (*n* = 103)	36	11	2	ND	4	ND	ND	38	41	52
Villegas et al. 2016 [36]	% prevalence of carbapenemase-producing Enterobacteriaceae from blood samples (*n* = 20)	ND	ND	ND	20 ^c^	ND	ND	ND	ND	ND	ND
Nicaragua											
Mayatepek et al. 1993 [29]	% resistance in *E. coli* isolates from diarrhea (ND)	94	16	ND	ND	56	98	ND	18	4	ND
Matute et al. 2004 [34]	% resistance in *E. coli* isolates from urine samples (UTI) (*n* = 35)	11	74	58	0	ND	ND	0	63	ND	30
Bours et al. 2010 [33]	% resistance in *E. coli* from urine samples (UTI) (*n* = 44)	25	61	46	ND	ND	ND	7	39	ND	32
Amaya et al. 2011 [27]	% resistance in *E. coli* isolates from stool samples (*n* = 727)	3 ^b^	60	ND	0	11	ND	ND	64	ND	3 ^b^
Amaya et al. 2012 [44]	% resistance in *E. coli* isolates from a hospital sewage sample (*n* = 32)	69	100	100	ND	97	ND	ND	100	ND	9
Hasan et al. 2016 [45]	% prevalence of ESBL-producing organisms from human stool (*n* = 28)	ND	ND	27	ND	ND	ND	ND	ND	ND	ND
Costa Rica											
Williams et al. 2003 [35]	% resistance in *E. coli* isolates from outpatient urine samples (*n* = 171)	ND	ND	ND	ND	ND	ND	8	40	ND	11
Rodríguez et al. 2006 [43]	% resistance in Enterobacteriaceae isolates from lettuce (ND)	100	ND	ND	ND	ND	ND	ND	ND	100	ND
Pérez et al. 2010 [30]	% resistance in pathenogenic *E. coli* strains from diarrhea (*n* = 52)	ND ^a^	40	11	ND	ND	ND	0	13	11	0
Jiménez et al. 2015 [39]	% resistance in *Salmonella* spp. isolates from gecko gut content (ND)	50	0	0	ND	0	ND	0	50	0	0
Molina et al. 2016 [41]	% resistance in *Salmonella* spp. isolates from animal feed (*n* = 110)	ND	ND	ND	ND	ND	ND	ND	ND	7	ND
Baldi et al. 2019 [40]	% resistance in *Salmonella* spp. isolates from raccoon fecal samples (*n* = 42)	ND	ND	ND	ND	ND	ND	ND	ND	ND	7
Pérez-Corrales et al. 2019 [31]	% resistance in enteroaggregative *E. coli* isolates from diarrhea (*n* = 189)	ND	54	ND	ND	ND	ND	ND	34	ND	0

Resistance rates are only shown for the most prevalent species as indicated in the Measure column, though some studies tested multiple species for resistance. If multiple percentages were reported (i.e., resistance to two antibiotics within the same class was studied), the highest was used in this table. Green represents resistance percentages or prevalence <15%, yellow 15 to <50%, and red 50% or greater, while ND indicates that a study did not investigate resistance to this class of antimicrobials. Values were rounded to the nearest whole number. This table excludes three of the articles that did not report percentages or prevalence: Liebana et al. 2004 [37] and Pasteran et al. 2012 [38] (case reports), and Pehrsson et al. 2016 [46] (resistomes). ^a^ This article did study aminoglycosides, but the proportion was not clearly reported; ^b^ these values were reported as less than or equal to 2.6%; ^c^ this value may include hospital-acquired infections because data specific to Central America (Guatemala) did not separate blood stream infections caused by community-acquired infections, like urinary tract infections, from those that were hospital-acquired.

## Supplementary Materials

The following are available online at https://www.mdpi.com/1660-4601/17/20/7622/s1, Supplementary Text: Literature search details, Table S1: Characteristics of Central American Countries, Table S2: Factors assessed in synthesis of extracted papers, Table S3: Brief description of included studies on CA-ARE in Central America.

## References

[1] World Health Organization (2017). Global Priority List of Antibiotic-Resistant Bacteria to Guide Research, Discovery, and Development of New Antibiotics.

[2] Van Boeckel T.P., Pires J., Silvester R., Zhao C., Song J., Criscuolo N.G., Gilbert M., Bonhoeffer S., Laxminarayan R. (2019). Global trends in antimicrobial resistance in animals in low- and middle-income countries. Science.

[3] World Health Organization (2015). Worldwide Country Situation Analysis: Response to Antimicrobial Resistance.

[4] Holmes A.H., Moore L.S.P., Sundsfjord A., Steinbakk M., Regmi S., Karkey A., Guerin P.J., Piddock L.J. (2016). Understanding the mechanisms and drivers of antimicrobial resistance. Lancet.

[5] Silbergeld E.K., Graham J., Price L.B. (2008). Industrial food animal production, antimicrobial resistance, and human health. Annu. Rev. Public Health.

[6] Graham J.P., Eisenberg J.N.S., Trueba G., Zhang L., Johnson T.J. (2017). Small-Scale Food Animal Production and Antimicrobial Resistance: Mountain, Molehill, or Something in-between?. Environ. Health Perspect..

[7] Frost I., Van Boeckel T.P., Pires J., Craig J., Laxminarayan R. (2019). Global geographic trends in antimicrobial resistance: The role of international travel. J. Travel. Med..

[8] Finley R.L., Collignon P., Larsson D.G.J., McEwen S.A., Li X.-Z., Gaze W.H., Reid-Smith R., Timinouni M., Graham D.W., Topp E. (2013). The scourge of antibiotic resistance: The important role of the environment. Clin. Infect. Dis..

[9] von Wintersdorff C.J.H., Penders J., van Niekerk J.M., Mills N.D., Majumder S., van Alphen L.B., Savelkoul P.H., Wolffs P.F. (2016). Dissemination of Antimicrobial Resistance in Microbial Ecosystems through Horizontal Gene Transfer. Front. Microbiol..

[10] Van Puyvelde S., Deborggraeve S., Jacobs J. (2018). Why the antibiotic resistance crisis requires a One Health approach. Lancet Infect. Dis..

[11] Lammie S.L., Hughes J.M. (2016). Antimicrobial Resistance, Food Safety, and One Health: The Need for Convergence. Annu. Rev. Food Sci. Technol..

[12] Collignon P.J., McEwen S.A. (2019). One Health—Its Importance in Helping to Better Control Antimicrobial Resistance. Trop. Med. Infect. Dis..

[13] Moreno P., Cerón A., Sosa K., Morales M., Grajeda L.M., Lopez M.R., McCraken J.P., Cordón-Rosales C., Palmer G.H., Call D.R. (2020). Availability of over-the-counter antibiotics in Guatemalan corner stores. PLoS ONE.

[14] Ramay B.M., Lambour P., Cerón A. (2015). Comparing antibiotic self-medication in two socio-economic groups in Guatemala City: A descriptive cross-sectional study. BMC Pharmacol. Toxicol..

[15] Alvarado Sosa J.L., Villatoro C.R. (2016). Resistencia bacteriana en infecciones del tracto urinario de origen comunitario. Rev. Med. Interma Guatem.

[16] Ramay B.M., Caudell M.A., Cordón-Rosales C., Archila L.D., Palmer G.H., Jarquin C., Moreno P., McCracken J.P., Rosenkrantz L., Amram O. (2020). Antibiotic use and hygiene interact to influence the distribution of antimicrobial-resistant bacteria in low-income communities in Guatemala. Sci. Rep..

[17] PAHO/WHO ReLAVRA. https://www.paho.org/hq/index.php?option=com_content&view=article&id=6221:2017-relavra-mas-informacion&Itemid=42432&lang=en.

[18] (2014). Annual Report of the Network for Monitoring/Surveillance of Antibiotic Resistance and Health Care Associated Infections—2014. Revista de Patologia Tropical. https://www.paho.org/hq/dmdocuments/2017/2014-cha-informe-anual-relavra.pdf.

[19] World Health Organization (2018). Global Antimicrobial Resistance Surveillance System (GLASS) Report: Early Implementation 2017–2018.

[20] World Health Organization (2020). Global Antimicrobial Resistance and Use Surveillance System (GLASS) Report: Early Implementation 2020.

[21] Plan de Accion Nacional de Lucha Contra la Resistencia a los Antimicrobianos Costa Rica 2018–2025. https://www.ministeriodesalud.go.cr/index.php/vigilancia-de-la-salud/normas-protocolos-y-guias/resistencia-microbiana/3811-plan-de-accion-nacional-de-lucha-contra-la-resistencia-a-los-antimicrobianos-costa-rica-2018-2025/file.

[22] Moher D., Liberati A., Tetzlaff J., Altman D.G., PRISMA Group (2009). Preferred reporting items for systematic reviews and meta-analyses: The PRISMA statement. PLoS Med..

[23] Covidence. http://www.covidence.org.

[24] Paterson D.L. (2006). Resistance in gram-negative bacteria: Enterobacteriaceae. Am. J. Med..

[25] Carattoli A. (2009). Resistance plasmid families in Enterobacteriaceae. Antimicrob. Agents Chemother..

[26] Bush K. (2010). Alarming β-lactamase-mediated resistance in multidrug-resistant Enterobacteriaceae. Curr. Opin. Microbiol..

[27] Amaya E., Reyes D., Vilchez S., Paniagua M., Möllby R., Nord C.E., Weintraub A. (2011). Antibiotic resistance patterns of intestinal Escherichia coli isolates from Nicaraguan children. J. Med. Microbiol..

[28] Mata L.J., Gangarosa E.J., Cáceres A., Perera D.R., Mejicanos M.L. (1970). Epidemic Shiga bacillus dysentery in Central America. I. Etiologic investigations in Guatemala, 1969. J. Infect. Dis..

[29] Mayatepek E., Seebass E., Hingst V., Kroeger A., Sonntag H.G. (1993). Prevalence of enteropathogenic and enterotoxigenic Escherichia coli in children with and without diarrhoea in Esteli, Nicaragua. J. Diarrhoeal. Dis. Res..

[30] Pérez C., Gómez-Duarte O.G., Arias M.L. (2010). Diarrheagenic Escherichia coli in children from Costa Rica. Am. J. Trop. Med. Hyg..

[31] Pérez-Corrales C., Leandro-Sandí K. (2019). Diarrheagenic Escherichia coli in Costa Rican children: A 9-year retrospective study. BMC Res. Notes.

[32] Shears P., Hart C.A., Suliman G. (1988). A preliminary investigation of antibiotic resistance in Enterobacteriaceae isolated from children with diarrhoea from four developing countries. Ann. Trop. Med. Parasitol..

[33] Bours P.H.A., Polak R., Hoepelman A.I.M., Delgado E., Jarquin A., Matute A.J. (2010). Increasing resistance in community-acquired urinary tract infections in Latin America, five years after the implementation of national therapeutic guidelines. Int. J. Infect. Dis..

[34] Matute A.J., Hak E., Schurink C.A.M., McArthur A., Alonso E., Paniagua M., Van Asbeck E., Roskott A.M., Froeling F., Rozenberg-Arska M. (2004). Resistance of uropathogens in symptomatic urinary tract infections in León, Nicaragua. Int. J. Antimicrob. Agents.

[35] Williams D.N., Sannes M.R., Eckhoff A.A., Peterson P.K., Johnson J.R., Sannes M.R. (2003). Antimicrobial resistance in Escherichia coli causing urinary tract infections in Costa Rica: A clinical dilemma. Int. J. Antimicrob. Agents.

[36] Villegas M.V., Pallares C.J., Escandón-Vargas K., Hernández-Gómez C., Correa A., Álvarez C., Rosso F., Matta L., Luna C., Zurita J. (2016). Characterization and Clinical Impact of Bloodstream Infection Caused by Carbapenemase-Producing Enterobacteriaceae in Seven Latin American Countries. PLoS ONE.

[37] Liebana E., Batchelor M., Torres C., Briñas L., Lagos L.A., Abdalhamid B., Hanson N.D., Martinez-Urtaza J. (2004). Pediatric infection due to multiresistant Salmonella enterica serotype Infantis in Honduras. J. Clin. Microbiol..

[38] Pasteran F., Albornoz E., Faccone D., Gomez S., Valenzuela C., Morales M., Estrada P., Valenzuela L., Matheu J., Guerriero L. (2012). Emergence of NDM-1-producing Klebsiella pneumoniae in Guatemala. J. Antimicrob. Chemother..

[39] Jiménez R.R., Barquero-Calvo E., Abarca J.G., Porras L.P. (2015). Salmonella Isolates in the Introduced Asian House Gecko (*Hemidactylus frenatus*) with Emphasis on Salmonella Weltevreden, in Two Regions in Costa Rica. Vector. Borne Zoonotic. Dis..

[40] Baldi M., Barquero Calvo E., Hutter S.E., Walzer C. (2019). Salmonellosis detection and evidence of antibiotic resistance in an urban raccoon population in a highly populated area, Costa Rica. Zoonoses Public Health.

[41] Molina A., Granados-Chinchilla F., Jiménez M., Acuña-Calvo M.T., Alfaro M., Chavarría G. (2016). Vigilance for Salmonella in Feedstuffs Available in Costa Rica: Prevalence, Serotyping and Tetracycline Resistance of Isolates Obtained from 2009 to 2014. Foodborne Pathog. Dis..

[42] Jarquin C., Alvarez D., Morales O., Morales A.J., López B., Donado P., Valencia M.F., Arevalo A., Munoz F., Walls I. (2015). Salmonella on Raw Poultry in Retail Markets in Guatemala: Levels, Antibiotic Susceptibility, and Serovar Distribution. J. Food Prot..

[43] Rodríguez C., Lang L., Wang A., Altendorf K., García F., Lipski A. (2006). Lettuce for Human Consumption Collected in Costa Rica Contains Complex Communities of Culturable Oxytetracycline- and Gentamicin-Resistant Bacteria. Appl. Environ. Microbiol..

[44] Amaya E., Reyes D., Paniagua M., Calderón S., Rashid M.-U., Colque P., Kühn I., Möllby R., Weintraub A., Nord C.E. (2012). Antibiotic resistance patterns of Escherichia coli isolates from different aquatic environmental sources in León, Nicaragua. Clin. Microbiol. Infect..

[45] Hasan B., Laurell K., Rakib M.M., Ahlstedt E., Hernandez J., Caceres M., Järhult J.D. (2016). Fecal Carriage of Extended-Spectrum β-Lactamases in Healthy Humans, Poultry, and Wild Birds in León, Nicaragua-A Shared Pool of blaCTX-M Genes and Possible Interspecies Clonal Spread of Extended-Spectrum β-Lactamases-Producing Escherichia coli. Microb. Drug Resist..

[46] Pehrsson E.C., Tsukayama P., Patel S., Mejía-Bautista M., Sosa-Soto G., Navarrete K.M., Calderon M., Cabrera L., Hoyos-Arango W., Bertoli M.T. (2016). Interconnected microbiomes and resistomes in low-income human habitats. Nature.

[47] Committee for Medicinal Products for Veterinary Use, Committee for Medicinal Products for Human Use (2019). Categorisation of antibiotics in the European Union.

[48] Mitchell M.E.V., Alders R., Unger F., Nguyen-Viet H., Le T.T.H., Toribio J.-A. (2020). The challenges of investigating antimicrobial resistance in Vietnam—What benefits does a One Health approach offer the animal and human health sectors?. BMC Public Health.

[49] Rüegg S.R., Nielsen L.R., Buttigieg S.C., Santa M., Aragrande M., Canali M., Ehlinger T., Chantziaras I., Boriani E., Radeski M. (2018). A Systems Approach to Evaluate One Health Initiatives. Front. Vet. Sci..

[50] Rozwandowicz M., Brouwer M.S.M., Fischer J., Wagenaar J.A., Gonzalez-Zorn B., Guerra B., Mevius D.J., Hordijk J. (2018). Plasmids carrying antimicrobial resistance genes in Enterobacteriaceae. J. Antimicrob. Chemother..

[51] Lynch J.P., Clark N.M., Zhanel G.G. (2013). Evolution of antimicrobial resistance among Enterobacteriaceae (focus on extended spectrum β-lactamases and carbapenemases). Expert Opin. Pharmacother..

[52] Bonelli R.R., Moreira B.M., Picão R.C. (2014). Antimicrobial resistance among Enterobacteriaceae in South America: History, current dissemination status and associated socioeconomic factors. Drug Resist. Update.

