# Supplementary material for: Community-Acquired Antimicrobial Resistant Enterobacteriaceae in Central America: A One Health Systematic Review
Source: Int J Environ Res Public Health. 2020 Oct 19;17(20):7622. doi: 10.3390/ijerph17207622 (PMC7589814; doi:10.3390/ijerph17207622)
Supplement: Supplementary file 1 [file ijerph-17-07622-s001.pdf]

International Journal of *Environmental Research and Public Health*

Supplementary Material:

Literature search details

PubMed search was conducted February 10, 2020 with the following terms:

(“antimicrobia*” OR “antibiotico” OR “antibiotic” OR “antibiotics” OR “anti-microbia*” OR “anti-biotic*” OR “antimicrobial drug resistance”[MeSH] OR “drug resistance, microbial”[MeSH] OR "anti-bacterial agents"[MeSH] OR “extended spectrum” OR “ESBL” OR “carbapenem” OR “colistin” OR “MRSA” OR “methicillin resistant” AND (“Central America” OR “Guatemala” OR “Panama” OR “Nicaragua” OR “Honduras” OR “Costa Rica” OR “El Salvador” OR “Belize”))

Table S1. Characteristics of Central American Countries.

Country	Population [a]	Life Expectancy [b]	Infant Mortality [c]	Basic Water Access [d]	Basic Sanitation Access [e]	Gross National Income (GNI) [f]	Income Classification [g]
Belize	0.390	74.5	11.2	98	88	4,450	Upper Middle
Costa Rica	5.05	80.1	7.6	>99	98	11,700	Upper Middle
El Salvador	6.45	73.1	11.8	97	87	4,000	Lower Middle
Guatemala	16.6	74.1	22.1	94	65	4,610	Upper Middle
Honduras	9.75	75.1	15.1	95	81	2,390	Lower Middle
Nicaragua	6.55	74.3	15.7	82	74	1,910	Lower Middle
Panama	4.25	78.3	13.1	96	83	14,950	High

[a] In millions, World Bank 2019 Indicator [1]; [b] At birth (years), World Bank 2018 Indicator [1]; [c] Per 1000 live births, World Bank 2018 Indicator [1].; [d] Percent of national population with access to improved water sources within 30 minutes round trip collection time, WHO/UNICEF Joint Monitoring Programme 2017 estimates [2]; [e] Percent of national population with access to improved sanitation facilities that are not shared with other households, WHO/UNICEF Joint Monitoring Programme 2017 estimates [2].; [f] Per capita using Atlas method (current US$), World Bank 2019 Indicator [1].; [g] Income classification based on GNI per capita (current US$) calculated using the Atlas method [3].

Table S2. Factors assessed in synthesis of extracted papers.

- Authors
- Year published
- Journal
- Data collection timeframe
- Study Title
- Country
- Urban or Rural location
- Specimen type (human/animal)
- Animal type
- Sample size (human/animal)
- Study design
- Species studied
- Types of resistance studied
- Antibiotic used
- Molecular methods
- Epidemiological methods (to determine risk)
- Laboratory method for typing
- Key findings

Table S3. Brief description of included studies on CA-ARE in Central America [a].

Authors	Location and/or Country	Sample Source and Size	Bacterial Species Studied	Phenotypic Resistance Studied [b]
Shears et al. 1988 [4]	San Pedro, Belize	Children w/ diarrhea (n = 9)	Enterobacteriaceae	Amp, Chl, Str, Sul, Tet, Tmp
Williams et al. 2003 [5]	San José, Costa Rica	Adults w/ urinary tract infection (unspecified n)	*Escherichia coli*	Cip, Nit, Sxt
Rodríguez et al. 2006 [6]	Costa Rica (2 cities)	Lettuce (n = 30)	Enterobacteriaceae	Gen, Otc
Pérez et al. 2010 [7]	Costa Rica	Children w/ diarrhea (n = 173)	*Escherichia coli*	Amc, Amp, Car, Cfz, Cro, Cxm, Cef, Cip, Gen, Lvx, Min, Nal, Nit, Nor, Tim, Tob, Sxt
Jiménez et al. 2015 [8]	Costa Rica	Asian house geckos (n = 115)	*Salmonella* spp.	Amp, Ctx, Caz, Chl, Cip, Nal, Nit, Pip, Ptz, Str, Sul, Tet, Sxt
Molina et al. 2016 [9]	Costa Rica	Animal feed (n = 1725)	*Salmonella* spp.	Tet
Baldi et al. 2019 [10]	Costa Rica (3 cities)	Raccoons (n = 86)	*Salmonella* spp.	Cip, Nal
Pérez-Corrales et al. 2019 [11]	San José, Costa Rica	Children w/ diarrhea (n = 12247)	*Escherichia coli*	Amp, Cip, Sxt
Pehrsson et al. 2016 [12]	El Salvador	Adults & children (n = 115) Environmental samples (n = 209)	Not applicable (resistome-based study)	Amo, Pen, Pip, Ptz, Fox, Ctx, Caz, Fep, Mem, Atm, Chl, Tet, Tgc, Gent, Cip, Col, Nit
Mata et al. 1970 [13]	Guatemala	Adults & children w/ & w/o diarrhea (n = 403)	*Shigella dysenteriae*, *Salmonella* spp.	Amp, Cef, Chl, Col, Ery, Gen, Kan, Nal, Neo, Nit, Pen, Str, Sul, Tet
Pasteran et al. 2012 [14]	Guatemala City, Guatemala	Adult (n = 1) Child (n = 1)	*Klebsiella pneumoniae*	Sxt, Min, Cip, Gen, Chl, Amk, Nal, Lvx, Tgc, Col, Fof, metallo-b-lactamase production, beta-lactams
Jarquín et al. 2015 [15]	Guatemala	Poultry products (n = 300)	*Salmonella* spp.	Amp, Amc, Cft, Chl, Cro, Enr, Gen, Str, Tet, Sxt

Villegas et al. 2016 [16]	Guatemala	Adults w/ blood stream infection (n = 20)[c]	Enterobacteriaceae	Carbapenems (carbapenemase-production)
Liebana et al. 2004 [17]	Comayagua, Honduras	Child (n = 1)	*Salmonella enterica*	Amk, Amp, Amc, Atm, Cft, Cxm, Caz, Ctx, Cro, Cfp, Fox, Cpd, Chl, Cls, Fur, Gen, Ipm, Nal, Neo, Str, Sxt, Tet, Tss
Mayatepek et al. 1993 [18]	Estelí, Nicaragua	Children w/ diarrhea (n = 50)<br>Children w/o diarrhea (n = 50)	*Escherichia coli*	Amp, Chl, Col, Gen, Kan, Tet, Sxt
Matute et al. 2004 [19]	León, Nicaragua	Adults w/ urinary tract infection (n = 208)	*E. coli, Klebsiella* spp., *Enterobacter* spp.	Amo, Amc, Cro, Caz, Cip, Cef, Gen, Mem, Nit, Nor, Sxt
Bours et al. 2010 [20]	León, Nicaragua	Adults w/ urinary tract infection (n = 304)	*E. coli, Serratia* spp., *E. fergusonii, Enterobacter* spp., *Klebsiella* spp.	Amk, Amp, Amc, Cef, Cro, Cip, Gen, Nit, Sxt
Amaya et al. 2011 [21]	León, Nicaragua	Children w/ diarrhea (n = 511)<br>Children w/o (n = 215)	*Escherichia coli*	Amp, Amc, Caz, Cro, Chl, Cip, Gen, Ipm, Sxt
Amaya et al. 2012 [22]	León, Nicaragua	Sewage & well-water (n = 118)	*Escherichia coli*	Amp, Amc, Ctx, Caz, Cro, Cip, Chl, Gen, Nal, Sxt
Hasan et al. 2016 [23]	León, Nicaragua	Poultry (n = 100)<br>Wild birds (n = 100)<br>Humans (n = 100)	*E. coli, Klebsiella pneumoniae*	Cephalosporins

[a] Information only relevant to Central America, results from isolates outside this geographic scope in the same article were excluded. [b] Antimicrobial abbreviations: Amc = amoxicillin-clavulanic acid, Amk = amikacin, Amo = amoxicillin, Amp = ampicillin, Atm = aztreonam, Car = carbenicillin, Caz = ceftazidime, Cef = cephalothin, Chl = chloramphenicol, Cfp = cefoperazone, Cft = ceftiofur, Cfz = cefazolin, Cls = colistin sulfate, Cpd = cefpodoxime, Cro = ceftriaxone, Col = colistin, Ctx = cefotaxime, Cxm = cefuroxime, Enr = enrofloxacin, Ery = erythromycin, Fep = cefepime, Fof = fosfomycin, Fox = cefoxitin, Fur = furazolidone, Gen = gentamicin, Ipm = imipenem, Kan = kanamycin, Lvx = levofloxacin, Mem = meropenem, Min = minocycline, Nal = nalidixic acid, Neo = neomycin, Nor = norfloxacin, Nit = nitrofurantoin, Otc = oxytetracycline, Pen = penicillin, Pip = piperacillin, Ptz = piperacillin-tazobactam, Str = streptomycin, Sul = sulfathiazole, Sxt = trimethoprim-sulfamethoxazole, Tet = tetracycline, Tgc = tigecycline, Tim = ticarcillin-clavulanic acid, Tmp = trimethoprim, Tob = tobramycin, Tss = triple-sulfonamide solution. [c]Blood stream infections studied had bacteremia sources from community-acquired and hospital-acquired infections, but country specific data did not differentiate between bacteremia sources.

References

1. World Development Indicators|DataBank. Available online: https://databank.worldbank.org/source/world-development-indicators (accessed on 23 June 2020).
2. Progress on Household Drinking Water, Sanitation and Hygiene 2000–2017. In *Special Focus on Inequalities*; United Nations Children's Fund (UNICEF) and World Health Organization: New York, NY, USA, 2019. Available online: https://www.who.int/water_sanitation_health/publications/jmp-2019-full-report.pdf (accessed on 16 October 2020).
3. New Country Classifications by Income Level: 2019–2020. Available online: https://blogs.worldbank.org/opendata/new-country-classifications-income-level-2019-2020 (accessed on 10 August 2020).
4. Shears P, Hart CA, Suliman G. A preliminary investigation of antibiotic resistance in Enterobacteriaceae isolated from children with diarrhoea from four developing countries. Ann Trop Med Parasitol. 1988;82: 185–188.

5. Williams DN, Sannes MR, Eckhoff AA, Peterson PK, Johnson JR, Sannes MR, et al. Antimicrobial resistance in Escherichia coli causing urinary tract infections in Costa Rica: a clinical dilemma. Int J Antimicrob Agents. 2003; 79–81.
6. Rodríguez C, Lang L, Wang A, Altendorf K, García F, Lipski A. Lettuce for Human Consumption Collected in Costa Rica Contains Complex Communities of Culturable Oxytetracycline- and Gentamicin-Resistant Bacteria. Appl Environ Microbiol. 2006;72: 5870–5876.
7. Pérez C, Gómez-Duarte OG, Arias ML. Diarrheagenic Escherichia coli in children from Costa Rica. Am J Trop Med Hyg. 2010;83: 292–297.
8. Jiménez RR, Barquero-Calvo E, Abarca JG, Porras LP. Salmonella Isolates in the Introduced Asian House Gecko (Hemidactylus frenatus) with Emphasis on Salmonella Weltevreden, in Two Regions in Costa Rica. Vector Borne Zoonotic Dis. 2015;15: 550–555.
9. Molina A, Granados-Chinchilla F, Jiménez M, Acuña-Calvo MT, Alfaro M, Chavarría G. Vigilance for Salmonella in Feedstuffs Available in Costa Rica: Prevalence, Serotyping and Tetracycline Resistance of Isolates Obtained from 2009 to 2014. Foodborne Pathog Dis. 2016;13: 119–127.
10. Baldi M, Barquero Calvo E, Hutter SE, Walzer C. Salmonellosis detection and evidence of antibiotic resistance in an urban raccoon population in a highly populated area, Costa Rica. Zoonoses Public Health. 2019;66: 852–860.
11. Pérez-Corrales C, Leandro-Sandí K. Diarrheagenic Escherichia coli in Costa Rican children: a 9-year retrospective study. BMC Res Notes. 2019;12: 297.
12. Pehrsson EC, Tsukayama P, Patel S, Mejía-Bautista M, Sosa-Soto G, Navarrete KM, et al. Interconnected microbiomes and resistomes in low-income human habitats. Nature. 2016;533: 212–216.
13. Mata LJ, Gangarosa EJ, Cáceres A, Perera DR, Mejicanos ML. Epidemic Shiga bacillus dysentery in Central America. I. Etiologic investigations in Guatemala, 1969. J Infect Dis. 1970;122: 170–180.
14. Pasteran F, Albornoz E, Faccone D, Gomez S, Valenzuela C, Morales M, et al. Emergence of NDM-1-producing Klebsiella pneumoniae in Guatemala. J Antimicrob Chemother. 2012;67: 1795–1797.
15. Jarquin C, Alvarez D, Morales O, Morales AJ, López B, Donado P, et al. Salmonella on Raw Poultry in Retail Markets in Guatemala: Levels, Antibiotic Susceptibility, and Serovar Distribution. J Food Prot. 2015;78: 1642–1650.
16. Villegas MV, Pallares CJ, Escandón-Vargas K, Hernández-Gómez C, Correa A, Álvarez C, et al. Characterization and Clinical Impact of Bloodstream Infection Caused by Carbapenemase-Producing Enterobacteriaceae in Seven Latin American Countries. PLoS One. 2016;11: e0154092.
17. Liebana E, Batchelor M, Torres C, Briñas L, Lagos LA, Abdalhamid B, et al. Pediatric infection due to multiresistant Salmonella enterica serotype Infantis in Honduras. J Clin Microbiol. 2004;42: 4885–4888.
18. Mayatepek E, Seebass E, Hingst V, Kroeger A, Sonntag HG. Prevalence of enteropathogenic and enterotoxigenic Escherichia coli in children with and without diarrhoea in Esteli, Nicaragua. J Diarrhoeal Dis Res. 1993;11: 169–171.
19. Matute AJ, Hak E, Schurink CAM, McArthur A, Alonso E, Paniagua M, et al. Resistance of uropathogens in symptomatic urinary tract infections in León, Nicaragua. Int J Antimicrob Agents. 2004;23: 506–509.
20. Bours PHA, Polak R, Hoepelman AIM, Delgado E, Jarquin A, Matute AJ. Increasing resistance in community-acquired urinary tract infections in Latin America, five years after the implementation of national therapeutic guidelines. Int J Infect Dis. 2010;14: e770-4.
21. Amaya E, Reyes D, Vilchez S, Paniagua M, Möllby R, Nord CE, et al. Antibiotic resistance patterns of intestinal Escherichia coli isolates from Nicaraguan children. J Med Microbiol. 2011;60: 216–222.
22. Amaya E, Reyes D, Paniagua M, Calderón S, Rashid M-U, Colque P, et al. Antibiotic resistance patterns of Escherichia coli isolates from different aquatic environmental sources in León, Nicaragua. Clin Microbiol Infect. 2012;18: E347-54.
23. Hasan B, Laurell K, Rakib MM, Ahlstedt E, Hernandez J, Caceres M, et al. Fecal Carriage of Extended-Spectrum β-Lactamases in Healthy Humans, Poultry, and Wild Birds in León, Nicaragua-A Shared Pool of blaCTX-M Genes and Possible Interspecies Clonal Spread of Extended-Spectrum β-Lactamases-Producing Escherichia coli. Microb Drug Resist. 2016;22: 682–687.
